# Analysis of the Deterioration Mechanisms of Tools in the Process of Forging Elements for the Automotive Industry from Nickel–Chromium Steel in Order to Select a Wear-Limiting Coating

**DOI:** 10.3390/ma18010013

**Published:** 2024-12-24

**Authors:** Marzena M. Lachowicz, Jacek Ziemba, Marta Janik, Agnieszka Trusz, Marek Hawryluk

**Affiliations:** 1 Department of Metal Forming, Welding and Metrology, Wroclaw University of Science and Technology, Lukasiewicza Street 5, 50-370 Wroclaw, Poland; marzena.lachowicz@pwr.edu.pl (M.M.L.); jacek.ziemba@pwr.edu.pl (J.Z.); 2 MAHLE Polska, Mahle 6, 63-700 Krotoszyn, Poland; marta.janik@mahle.com; 3 Department of Air-Conditioning, Heating, Gas Engineering and Air Protection, Wroclaw University of Science and Technology, Plac Grunwaldzki 13, 50-377 Wrocław, Poland; agnieszka.trusz@pwr.edu.pl

**Keywords:** hot forging in closed dies, tool life, surface engineering techniques, hybrid layers, destructive mechanisms

## Abstract

This paper provides a detailed analysis of the operation of representative forging tools (in the context of using various surface engineering techniques) used in the process of the hot forging of nickel–chromium steel elements. The influence of the microstructure and hardness of the material on the durability of the tools is also discussed, which is important for understanding the mechanisms of their wear. The research showed that the standard tools used in the process (only after nitriding) as a reference point worked for the shortest period, making an average of about 1400 forgings. In turn, the tools coated with the CrAlSiN coating allowed for the production of the largest number of forgings, reaching 2400 pieces, with uniform wear. In comparison, the tools with the CrAlBN coating made 1900 forgings. Three-dimensional scanning analysis showed that CrAlSiN- and CrAlBN-coated tools have lower volumetric wear, around 41–43 mm^3^, compared to 59 mm^3^ for nitrided tools. For a better comparison of tool life, the authors proposed the Z-factor, as the material loss to the number of forgings. The CrAlSiN coating showed the lowest material loss, despite a slightly higher Z-factor value compared to the CrAlBN coating. The use of hybrid coatings such as CrAlSiN and CrAlBN significantly reduces tool wear while increasing service life compared to tools that are nitrided only.

## 1. Introduction

The wear of valves is one of the most important factors affecting the efficiency of a car engine [1]. Meanwhile, high and cyclic mechanical and thermal loads as well as high pressures expose the valves to extreme performance loads [2,3]. The valves are in contact with high temperatures exceeding 600 °C due to a large amount of heat released during fuel combustion [4]. Engine valve-type elements are usually made as forgings in processes of extrusion and forging at elevated temperatures from classic chromium–nickel or high-nickel austenitic steels as well as nickel superalloys (nickel content of about 80%). During the manufacturing of the technology of valve forgings from austenite steel (through extrusion and forging in closed dies), the obtained products characterize enhanced mechanical properties and surface finish compared to the relatively well-mastered technology of electro-upsetting. Unfortunately, forging-based technology is rarely applied as, despite the significant technological progress, it has not been mastered well enough because it requires ensuring the proper (in a narrow range) charge heating technology and designing the proper shapes of the tool impressions so that a complete and proper filling can be obtained. The biggest problem is, however, the elevated pressures, intense friction, and fluctuating temperatures, which create a variable and challenging tribological condition as well as increased adhesion, as a result of the increased adherence of the charge material to the base of a tool made of tool steel. In recent years, technological progress has been demanded so that an engine valve can characterize higher and higher durability as well as wear resistance at high temperatures, while the wear and failures of the valves have become the key problems [4]. This is determined by the existing regulations referring to combustion gas and the requirements concerning reduced fuel consumption, which increase the combustion temperature [5,6]. Experience shows that classic austenitic steels are becoming insufficient to fulfil those requirements. On the one hand, this has led to studies concentrating on valve protection [4]. On the other hand, new steel grades are searched for, and one of such proposed materials is nickel–chromium steel NCF3015 [6,7,8,9,10]. The use of this precipitation-hardened steel for valves generates additional problems during the forging process. One of the basic factors affecting the behavior of steel during forging is its microstructure determined by its chemical composition. The intermetallic phases present in it reduce its deformability and, at the same time, their hard particles lead to the accelerated wear of the tool [7,11,12]. All this translates to a significant (by a few times) reduction in forging tool durability during the production of steel forgings, especially compared to forging from typical carbon steels [13]. Low tool durability has been the subject of intensive studies for years, with the aim of increasing it [14].

The working surfaces of tools used in die forging processes at elevated temperatures are critical areas, whose state can significantly translate to the durability of the whole tool. A lower tool durability translates, in turn, to higher costs of the given production process, which leads to a search for methods of its increase. There is, however, a lack of defined criteria for selecting methods of improving the durability of forging dies and punches. We know the general guidelines and directions to be followed while searching for effective methods and techniques of increasing the performance time of the forging instrumentation. Each process should be considered individually since its parameters are directly influenced by the technology, the tribological conditions between the forged material and the tool, and numerous other factors are strictly related and dedicated to each process. The most popular currently applied durability increasing methods include the following: choosing the tool material and its appropriate heat treatment, optimizing the tool design, as well as methods related to surface engineering (including hybrid techniques, thermo-chemical treatments, welding, and mechanical processes), along with other methods not directly related to the die or punch [15].

A popular method for forging equipment aiming to increase its durability is thermo-chemical heat treatment, namely nitriding. This includes both gas nitriding and glow discharge (plasma) nitriding [16,17,18]. The less common methods include nitriding in fluid beds, nitriding in powders, and ion nitriding (Plasma Diffusion Treatment), e.g., the ZeroFlow method [19,20]. Regardless of the nitriding technique, this makes it possible to increase the tools’ abrasion resistance, fatigue strength, and resistance to corrosion. The nitrided layer must characterize in a proper and coherent microstructure adequate to the assumed designation and requirements. Observations from various industrial forging processes using nitrided tools have demonstrated that this method can enhance their durability severalfold [21,22,23,24]. One of the important directions of the development of tool material surface engineering aiming to increase tool durability is the application of hybrid layers. Owing to the combination and synergy of different technologies, they make it possible to obtain such surface layer properties which are unachievable in the case of using those techniques separately [21,22,23,24,25,26].

Hybrid techniques belong to the most recent methods of modifying the properties of the surface layer. The most frequently used hybrid layers are combinations of nitrided layers with PVD or CVD coatings. The structural result of their production is a multi-layered system, consisting of a well-prepared substrate—the nitrided layer—and an appropriately selected coating applied to the substrate surface, a protective (anti-wear) adhesive coating. Many years of research have shown that the use of a nitrided layer does not sufficiently protect the surface layer from the destructive influences, such as cyclic changeable thermal shocks, intensive friction, or high mechanical loads. A certain improvement in this area is obtained through increasing its thickness [24]. However, for many applications, this has become insufficient. In hybrid technologies, the main task of the nitrided layer is to increase the substrate’s hardness and resistance to plastic deformations; however, it also constitutes a gradient transition layer for a much harder yet also much thinner CVD/PVD-type coating. This protects the hard PVD or CVD coating from losing its internal coherence to the substrate, mitigating the impact of external damaging factors during operation [27,28,29,30,31]. What is more, the coatings also reduce the friction coefficient and prevent material sticking but also favor the formation of compression stresses (instead of tensile) in the sub-surface layer of the tool. It should be emphasized that the tool’s durability is also significantly affected by the hardness of the processed material. This translates to both the selection of the chemical composition of the processed material [7] and the preheating temperature [10].

The aim of this study was to analyze the applied surface engineering techniques: nitriding and selecting hybrid layers deposited onto the forging tools used in the process of hot forging in closed dies to produce a valve forging assigned for motor truck engines. As mentioned above, the diversity of the conditions present during the specific technological processes makes it not always possible to translate the results obtained under laboratory conditions to real service life conditions. This requires each technological process to be approached individually. For this reason, in order to achieve the abovementioned goal, performance tests of the produced layers were carried out, followed by an evaluation of their wear under service life conditions.

## 2. Materials and Methods

The test material was three representative (for each of the three groups) forging tools (dies) used in the process of hot forging in closed dies. All the tools were made from hot work tool steel–chromium–molybdenum–vanadium steel W360, produced by Böhler. The chemical composition of this steel has been designed in such a way so that it ensures a higher strength and wear resistance at high temperatures than steel H13 [32,33]. The hardness of the substrate material of the examined die equaled 59 HRC. Its chemical composition, determined with the use of the GD OES method, is presented in Table 1.

**Figure 1:**
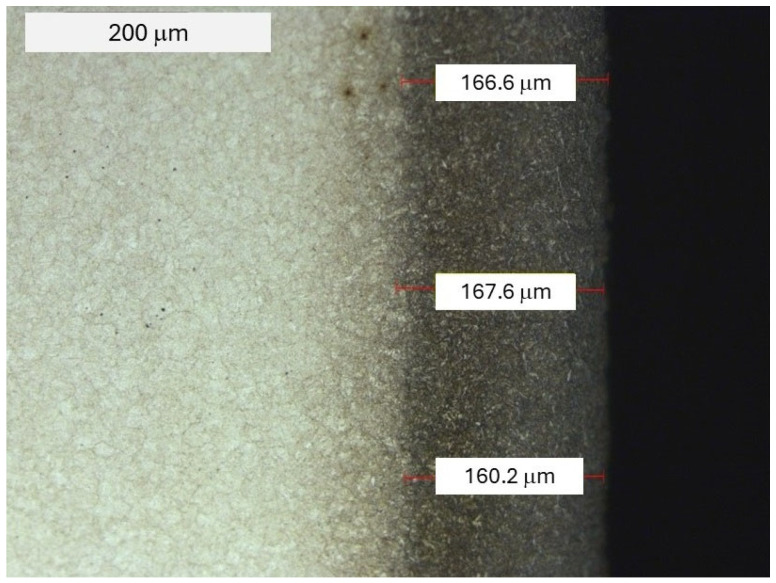
Microstructure of a die made from steel W360 with a deposited nitrided layer. Light microscopy and an etched state.

Among the three selected representative forging dies, the first one was constituted by a tool with a nitrided layer, which was at the same time the reference point for the other two tool groups, which, beside nitriding, were covered with two different coatings. Nitrided dies are a standard tool used in industrial processes. In turn, in order to increase durability, next to nitriding, CVD coatings were also applied. For the nitriding of all the tools, thermo-chemical treatment was carried out, consisting of nitriding. The following process parameters were selected: pressure: approx. 4 mbars, atmosphere: 10%N and 90%H, total process time: approx. 8 h, temperature: approx. 550 °C. The other two tools representative for the given group were covered with hybrid layers. The hybrid treatment included nitriding and the process of PVD coating deposition. In this way, the second die was obtained, with a nitrided layer and a PVD coating with the commercial name BIGAAN (CrAlBN), whereas the third one was made with a nitrided layer and a PVD coating with the commercial name ALWIN (CrAlSiN). Both coatings were produced by SHM (Czech Republic) in their own technology, which simultaneously applied two fundamentally diverse PVD methods [34], i.e., magnetron dusting and deposition with the use of a low-voltage electric arc. The coatings were deposited onto the tool according to the producer’s recommendations, and they constitute its “know-how”. The ALWIN coating, according to the producer’s data, is a nano-crystalline CrAlSiN coating with a high chromium content, used in applications requiring a high resistance to oxidation and sticking of the processed material to the tools. According to the manufacturer’s data, the coating thickness is 7–8.5 µm and the hardness is about 26–28 GPa. The BIGAAN coating is a patented CrAlBN coating, which makes it possible to obtain coatings with very high surface smoothness. The coating thickness is 3–5 µm and the hardness is 35 GPa. Figure 1 shows an exemplary microstructure for a tool after nitriding, which was typical for quenched and tempered materials.

**Figure 2:**
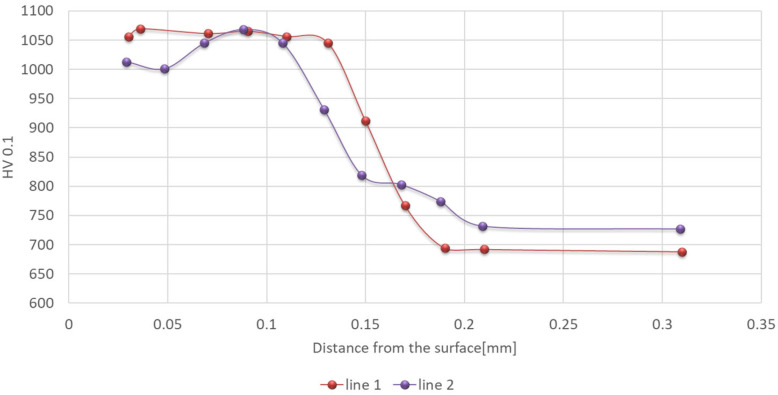
Hardness distribution in the sub-surface area for steel W360 after nitriding.

After nitriding, the material’s hardness in the sub-surface area exceeded 1000 HV0.1 (Figure 2). Based on the performed hardness distribution, we can state that the conventional thickness of the hardened layer determined on the basis of the hardness measurements (for the core hardness criterion of +50 HV) was in agreement with the value determined through light microscopy, which equaled about 166 µm.

For comprehensive analysis, the following research methods were employed:

– Macroscopic examination combined with measurement of wear/excess material on the tool’s working surface using a ROMER Absolute ARM 7520si (Hexagon Metrology, Stockholm, Sweden) integrated with an RS3 scanner, and comparison of the scan geometry with CAD models;
– Microstructure analysis of the surface layer in the tool’s cross-section performed with a Leica DM6000M microscope (Leica Microsystems, Wetzlar, Germany) after traditional metallographic sample preparation and etching with 5% nital (grinding and polishing on a Stuers350 (Struers S.A.S., Champigny sur Marne, France) grinder–polisher);
– Chemical composition analysis using glow discharge optical emission spectroscopy (GDOES) with a LECO GDS 500A analyzer (LECO Corporation, Michigan, USA);
– Observation of damage features on the working surface with a Phenom ProX scanning electron microscope (Thermo Fisher Scientific, Waltham, MA, USA) coupled with an EDX detector (Thermo Fisher Scientific, Waltham, MA, USA).

**Figure 3:**
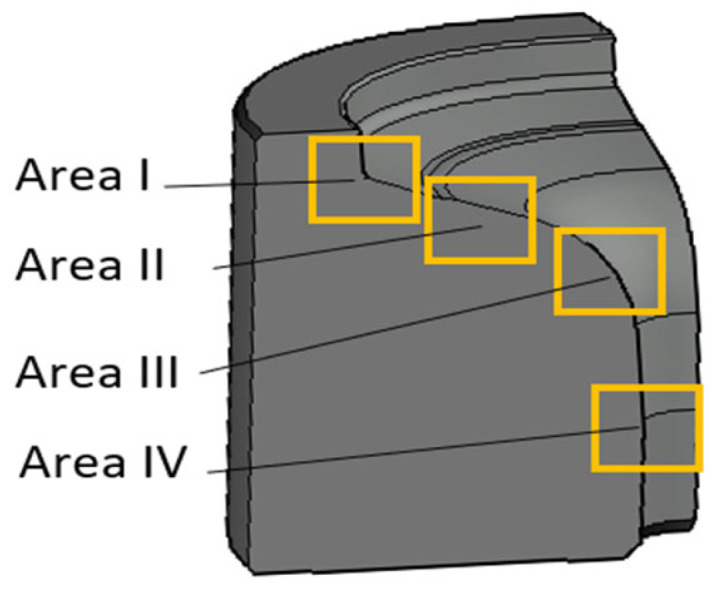
Scheme of microscopic observation areas.

The scheme of the microscopic observations is shown in Figure 3.

## 3. Results

### 3.1. Wear Analysis via 3D Scanning

**Figure 4:**
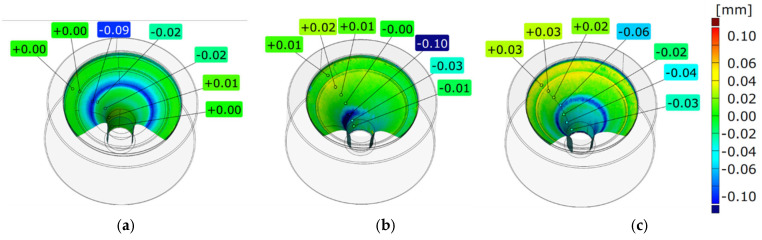
Results of 3D scanning of the selected tools at the end of their service life for a die covered with a layer: (**a**) nitrided, after 1520 forgings, (**b**) CrAlBN coating, after 1900 forgings, and (**c**) CrAlSiN coating, after 2400 forgings.

The three tools selected for the analysis, with visible traces of wear, produced the following number of forgings, respectively: nitrided—1520 forgings, CrAlBN coating—1900 forgings, and CrAlSiN coating—2400 forgings. These tools were subjected to 3D scanning with the use of the GOM Inspect Professional program. The tool scanning results showed degradation of the working surfaces in the form of a color map of deviations (Figure 4).

Based on the 3D scanning results presented in Figure 4, we can notice that the tools were made according to the assumptions for the nominal dimensions. On the basis of the performed tests, we can state the following:

(1) In the case of the nitrided tool (Figure 4a), which produced 1520 forgings, there is a visible stripe of blue wear (in zone II) in the form of a round stria in the upper part of the die, where the maximal wear value equals up to—0.04 mm. The analysis of a fragment of the surface of the upper part of zone II points to characteristic perpendicular lines proving material flow, while on the other remaining surfaces, there is no visible wear.
(2) In the case of the analysis of the tool covered with a CrAlBN coating (Figure 4b), which produced 1900 forgings, practically the whole tool surface does not exhibit wear beyond the local wear in the central part of the opening (in the upper part of zone III), visible in the form of a blue smudge, where the wear is higher than −0.1 mm and locally reaches up to −0.16. This type of wear proves the unpredictability of the wear process consisting of local wear.
(3) The third analyzed tool, covered with a CrAlSiN coating (Figure 4c), which produced 2400 forgings, exhibits uniform wear in the form of a blue ring (in the upper part of zone III). The depth of the coloration proves the occurrence of wear, where the maximal value of the deepening on this surface equals up to −0,06 mm and, in the remaining parts, deep into the tool (zone IV), on the narrowing, there are visible traces of non-uniform wear (loss of material) at the level of −0,04 mm. This tool, however, produced the highest number of forgings, with the predicted wear in those tool areas tolerating a change in the geometry of the produced forging detail.

**Figure 5:**
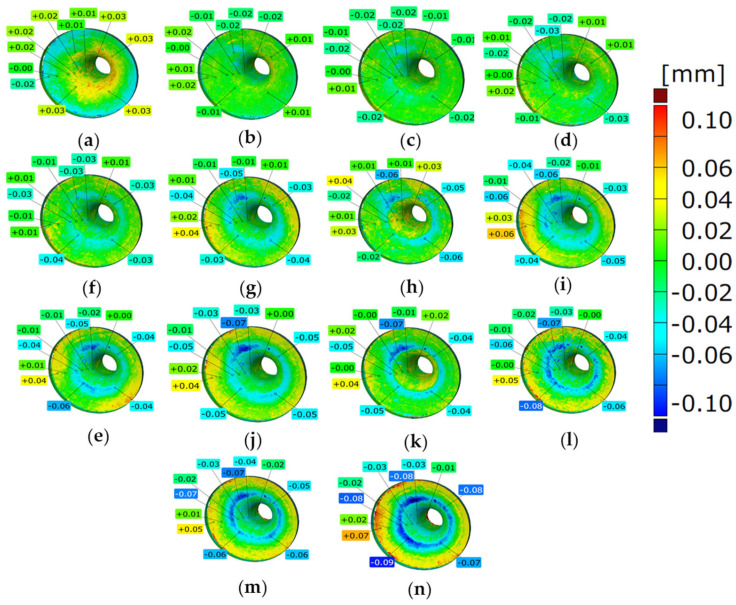
Three-dimensional scanning results for a forging cyclically collected from the process of forging a tool covered with a nitrided layer, showing the wear on the tool for the following forgings: (**a**) the 300th, (**b**) 400th, (**c**) 500th, (**d**) 600th, (**e**) 700th, (**f**) 800th, (**g**) 900th, (**h**) 1000th, (**i**) 1100th, (**j**) 1200th, (**k**) 1300th, (**l**) 1400th, (**m**) 1500th, and (**n**) 1520th.

In order to perform an analysis of the wear process during a single forging process, a decision was made to cyclically collect forgings, every 100 items, from the analyzed process for three tools. Figure 5 presents exemplary results from the 3D scanning of forgings collected from the process of forging a tool covered with a nitrided layer, which produced 1520 items. Based on the scanning of the forgings cyclically collected from the process, we can perform an analysis (service life history) of the changes in the tool which produced those forgings. The authors call studies of this type “reverse 3D scanning”.

**Figure 6:**
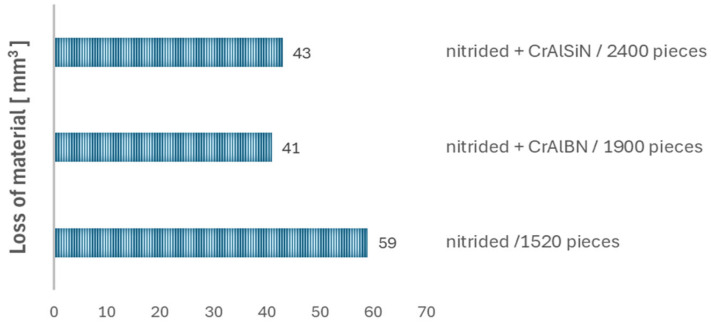
Results of volumetric wear calculated from the tools for a nitrided die after 1520 forgings, hybrid layer: nitrided + CrAlBN coating after 1900 forgings and hybrid layer: nitrided + CrAlSiN coating after 2400 forgings.

In the analysis of the results presented in Figure 5, we can notice that, up to the 700th forging, the wear is very insignificant and locally equals up to −0.03 mm. From the 700th forging up, there is wear visible on a fragment of the circumference in the form of a blue coloration, locally up to −0.05 mm, which expands up to the 1300th forging, where a second wear initiation occurs, more or less on the opposite side. From that moment on, the wear grows uniformly, forming a ring. For the last forging, the wear overlaps with the values measured for the tool. The performed total (global) analysis presented in Figure 6 makes it possible to notice that the biggest wear (loss of material) takes place for the nitrided layer as the obtained total loss was at the level of 59 mm^3^.

**Figure 7:**
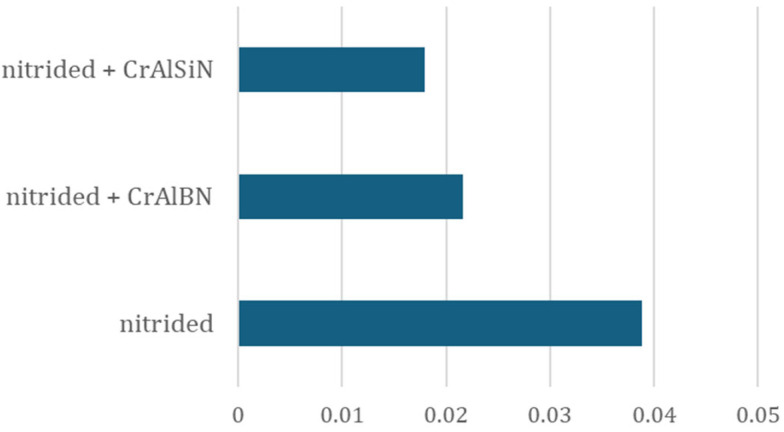
Relative wear coefficient Z determined for the tested dies.

In the case of the tools covered with the coatings (both CrAlSiN and CrAlBN coatings), the volumetric wear values are very comparable and equal about 41–43 mm^3^. In the analysis and comparison of all the results of the tests related to the 3D scanning of tools and forgings, it is worth mentioning that, among all the examined dies, the tool covered with the CrAlSiN coating made it possible to produce the highest number of forgings, and the character of its wear is predictable, similarly to the case of the nitrided tool. This demonstrates that the CrAlSiN coating has the best performance properties for the analyzed process. What is more, for an even more full analysis for the purposes of this study, the relative wear coefficient Z was also determined, understood as the loss of material referred to the number of elements forged by the tool (Figure 7).

Next, through a comparison of the material loss results and the value of coefficient Z, the conducted analysis makes it possible to state that, despite the slightly higher material loss value (Figure 6) compared to the CrAlBN coating, its lowest value is exhibited by the CrAlSiN coating. The highest value of the coefficient—about twice as high as that of the examined coatings—is demonstrated by the die without an additional CVD coating, that is only with a nitrided layer. This means that the use of coatings resulted in a two-fold increase in the unit wear of the die, understood as the wear occurring during the forging of a single tool.

### 3.2. Microscopic Tests

#### 3.2.1. Nitrided Die Tests

**Figure 8:**
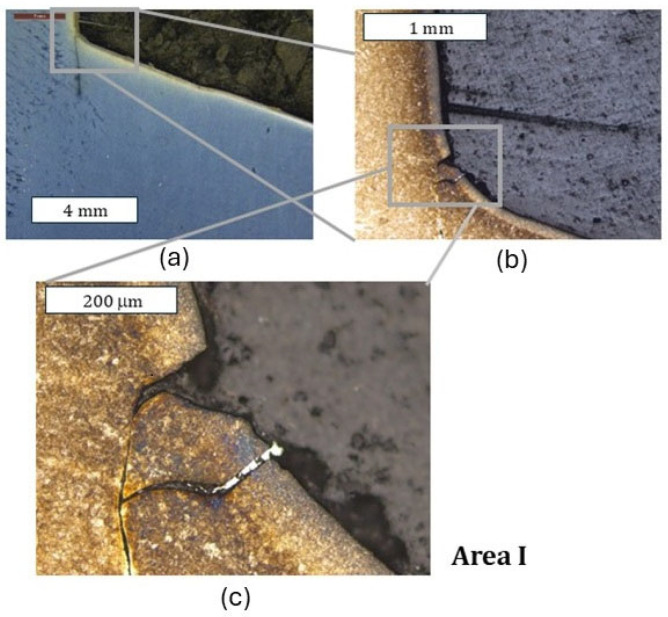
Macrostructure and microstructure of the die in zone I: (**a**) stereoscopic microscopy; (**b**,**c**) light microscopy. Etched state.

**Figure 9:**
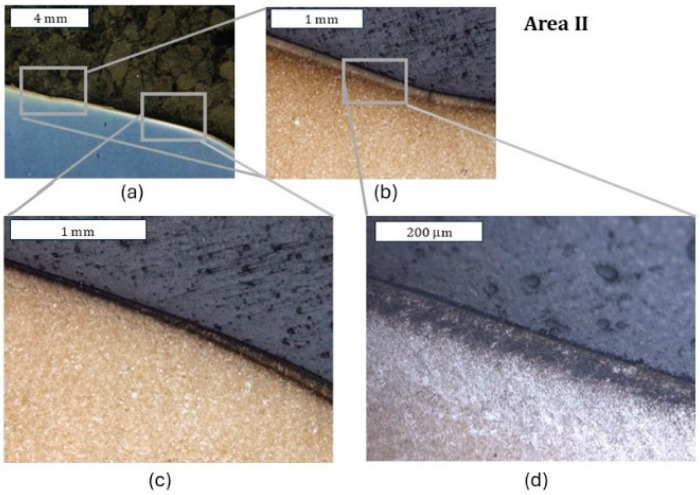
Macrostructure and microstructure of the die in zone II: (**a**) stereoscopic microscopy; (**b**–**d**) light microscopy. Visible reduction in the nitrided layer’s thickness caused by abrasion wear, leading to the formation of a narrowing on the die (**c**) and plastic deformation in the sub-surface area (**d**). Etched state.

**Figure 10:**
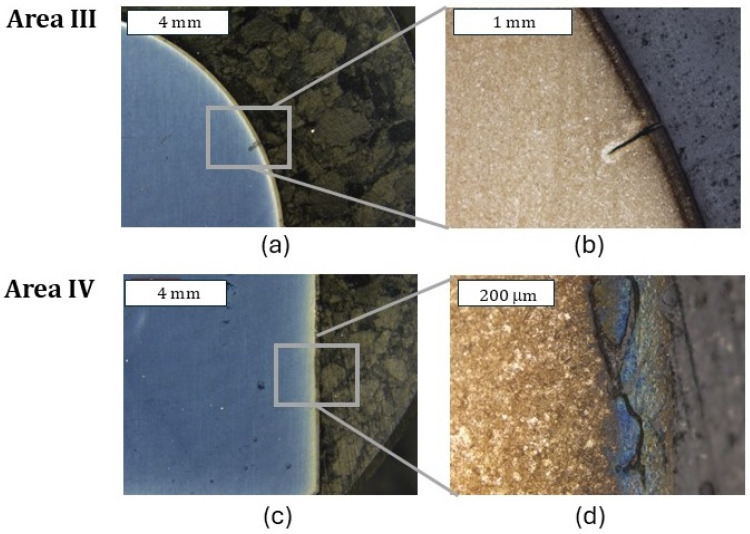
Macrostructure and microstructure of the die in zone III and IV: (**a**,**c**) stereoscopic microscopy; (**b**,**d**) light microscopy. Etched state.

As shown by the 3D scanning results, the die which was only nitrided characterized the highest loss of material. That loss was accompanied by numerous cracks in zone IV localized in the lower part of the die. The obtained results for this die have also been presented in a form showing the macrostructure of the tested elements, in order to facilitate the localization of the particular die areas. The cracks were forming in the areas of the presence of construction notches, such as the change in the cross-section occurring in zone I (Figure 8), or were perpendicular to the surface in zone III. The tests show that the thickness of the nitrided layer is of great importance for the durability of the die [24]. A reduced thickness of the nitrided layer in the upper part of the die (zone II) was also locally observed, which should be related to the abrasion wear observed also at the stage of 3D scanning (Figure 9). It is this area that is mainly responsible for the loss of mass of the die observed during 3D scanning. In zone II, plastic deformation was also observed in the sub-surface area (Figure 9d). This is also in agreement with the observations obtained from 3D scanning, where numerous flows were observed in this area. The biggest damage was recorded in the lower part of the die, in zone IV (Figure 10c,d). This was also accompanied by surface oxidation, which proves that, in this area, the thermal load was the highest. Despite that, in this area, no loss of material was observed during 3D scanning, which demonstrates that, despite the numerous sub-surface cracks, the die remained coherent. However, we should expect that, during its longer service life, a dramatic increase in mass loss will take place as a result of the spalling of material fragments.

#### 3.2.2. Tests of a Nitrided Tool with a CrAlBN Coating

**Figure 11:**
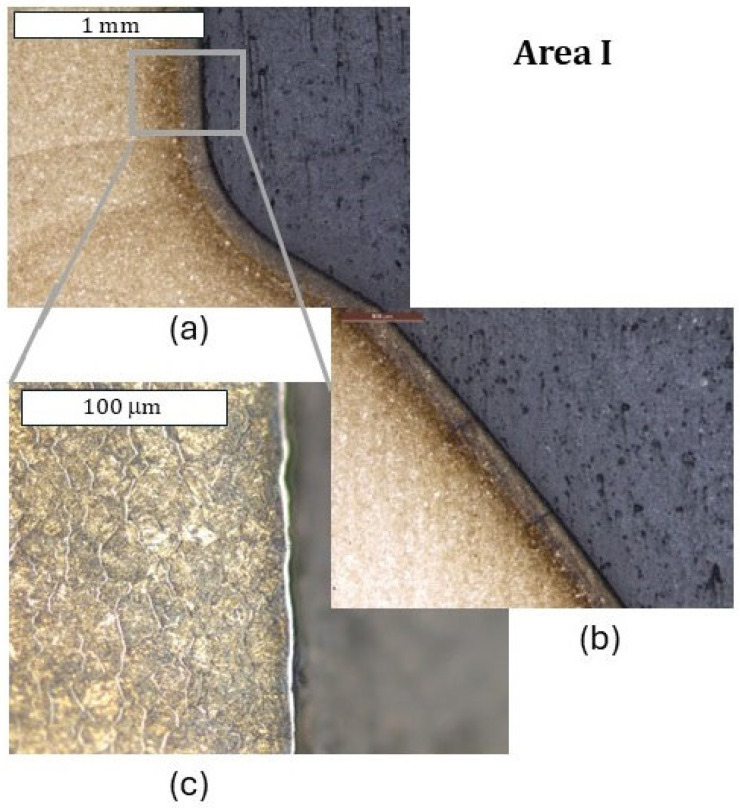
Microstructure of the die in two areas of zone I (**a**,**b**). Visible maintained continuity of the coating (**c**). Light microscopy. Etched state.

**Figure 12:**
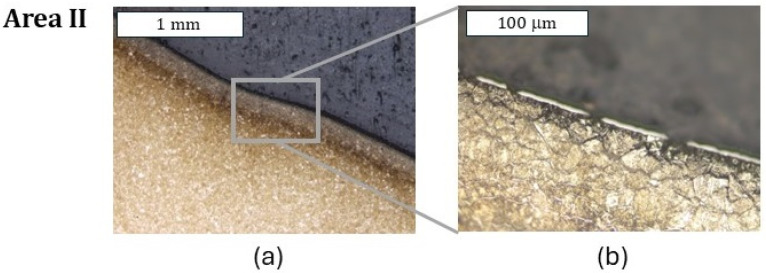
Microstructure of the material in zone II (**a**). Visible loss of coating continuity. (**b**) Light microscopy. Etched state.

**Figure 13:**
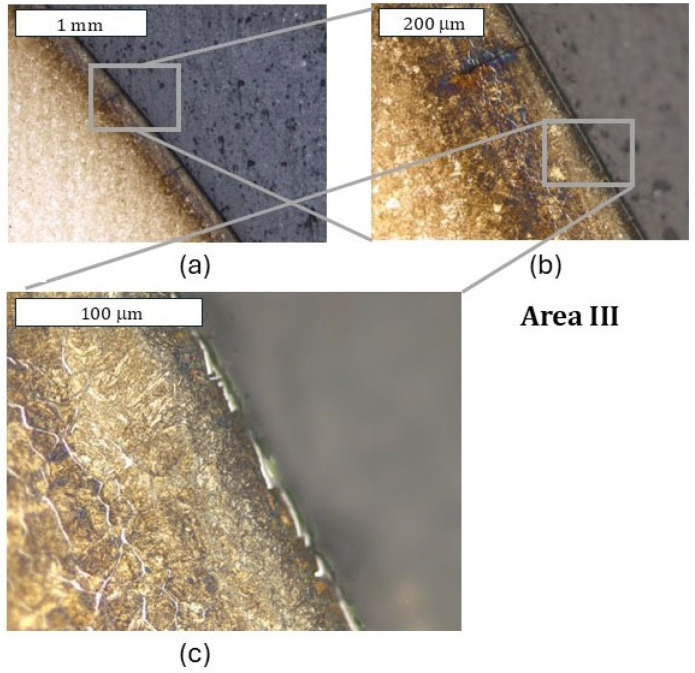
Microstructure of the material in zone III (**a**) and an enlarged fragment of this area (**b**). (**c**) Visible plastic deformation of the coating. Light microscopy. Etched state.

**Figure 14:**
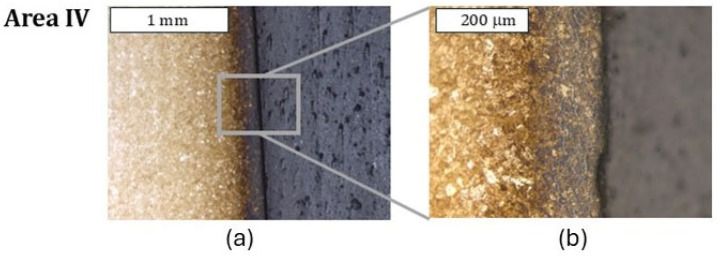
Microstructure of the material in zone IV (**a**) and an enlarged fragment of this area (**b**). Light microscopy. Etched state.

In an analogical zone I to that in the case of the previous tool (only with a nitrided layer) for the tool covered with a CrAlBN coating, only cracks perpendicular to the surface were observed, which did not go beyond the thickness of the nitrided layer (Figure 11). Because the deposited coating was of a micrometric size, it was possible to observe it only at larger magnifications; still, it demonstrated continuity (Figure 11c). In zone II, in which, for the nitrided layer, abrasion wear and plastic deformation were observed, only plastic deformation was visible (Figure 12). In this area, the coating demonstrated local tears, which led to material oxidation on the grain boundaries (Figure 12b). Liu et al. [35] showed that these coatings were accompanied by adhesive wear, which could be the reason for the loss of coating continuity in this area. The consequence of the loss of continuity was easier access of the forging heat to the substrate material. In zone III, plastic micro-deformations of the coating were observed (Figure 13). In the further section of the die, this zone transitioned into an area with more severe service conditions, in which cracks and surface oxidation were observed—zone IV (Figure 14). This led to the formation of surface chipping, which ultimately resulted in the uncontrolled wear observed in 3D scanning studies.

In this area, at larger magnifications, changes pointing to strong plastic deformation of the coating were recorded, which proves the significant effort of the material in this zone (Figure 13c). In this area, during 3D scanning, the presence of local wear was observed. It should be emphasized that it was impossible to collect samples for microscopic tests from the area of the highest wear. Based on the above observations, we should, however, expect that it was the cracks and the surface oxidations occurring at high surface loads that resulted in the formation of a “gap” in the material, which was recorded during 3D scanning. This was favored by the oxidation progressing along the grain boundaries. Such destruction mechanisms will favor the spalling of material fragments, which, ultimately, was the cause of a catastrophic destruction in the case of this die. No intensive wear in the remaining die zones, i.e., I, II, and IV, was observed, which was in agreement with the obtained scanning results. In the lower part of the die (zone IV), discolorations were also recorded, which proved the occurrence of surface oxidation; however, no visible surface spallings were noticed, as was the case of the nitrided-only die (Figure 10). This area was, however, totally void of the coating—it had probably undergone a thermo-mechanical removal—and the observations at larger magnifications point to the presence of microscopic surface spallings.

#### 3.2.3. Tests of a Nitrided Tool with a CrAlSiN Coating

**Figure 15:**
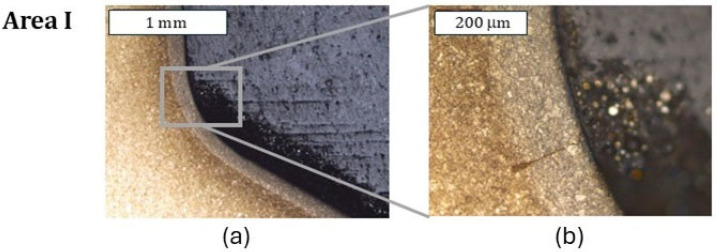
Microstructure of the material in zone I (**a**) and an enlarged fragment of this area (**b**). Light microscopy. Etched state.

**Figure 16:**
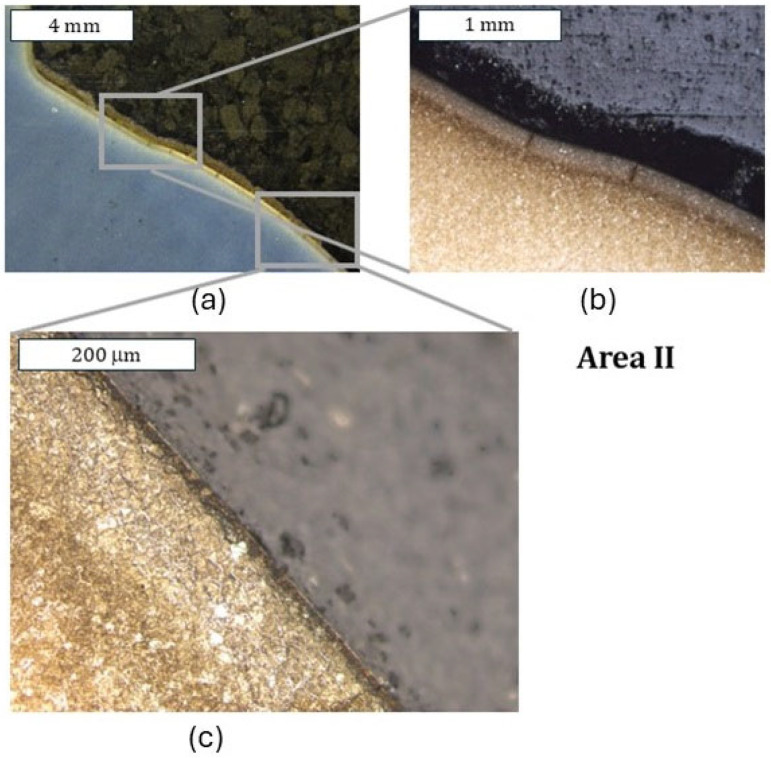
Macrostructure (**a**) and microstructure (**b**) of the die in zone II. Visible oxidation occurring on the grain boundaries and trace amounts of the CrAlSiN coating (**c**): (**a**) stereoscopic microscopy; (**b**,**c**) light microscopy. Etched state.

**Figure 17:**
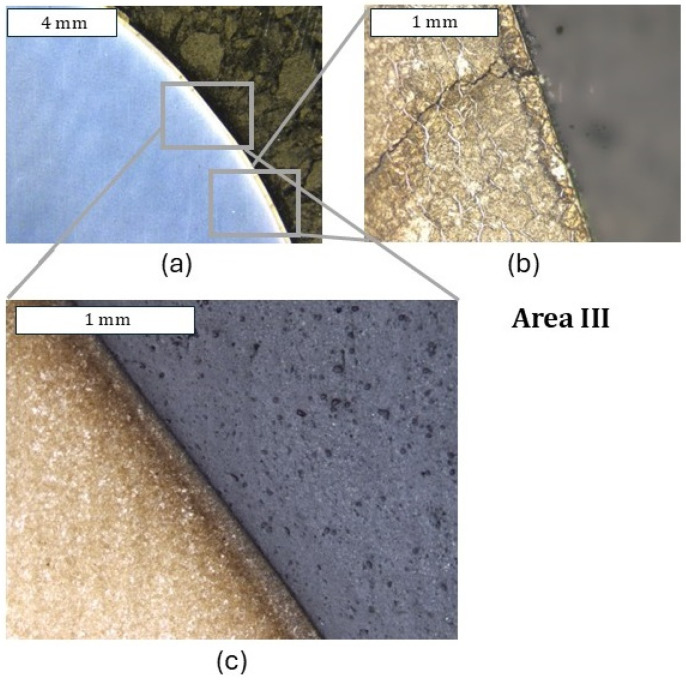
Macrostructure (**a**) and microstructure (**b**) of the die in zone III. Visible reduction in the nitrided layer’s thickness caused by abrasion wear, leading to the formation of a narrowing on the die (**c**); (**a**) stereoscopic microscopy; (**b**,**c**) light microscopy. Etched state.

Both tools (dies) covered with coatings in zone I exhibited a similar character of surface destruction, revealing itself through the formation of cracks perpendicular to the surface (Figure 15). Similarly to the case of the remaining dies, plastic deformation of the surface in zone II was observed, which, in particular, very well illustrates the macrostructure presented in Figure 16. In this area, trace amounts of the CrAlSiN coating were observed, with the coating having undergone mechanical degradation (Figure 16c). Also recorded was a local reduction in the nitrided layer’s thickness (Figure 17), which is in agreement with the 3D scanning results, showing the formation of a narrowing in zone III. It should be noted, however, that it was located lower than in the case of the nitrided-only die. In the latter’s case, that area was located in zone II. In zone III, numerous minor cracks in the sub-surface area were also recorded; however, they did not propagate beyond the thickness of the nitrided layer. Still, it is characteristic that the CrAlSiN coating in this area had been almost totally removed mechanically (Figure 17).

**Figure 18:**
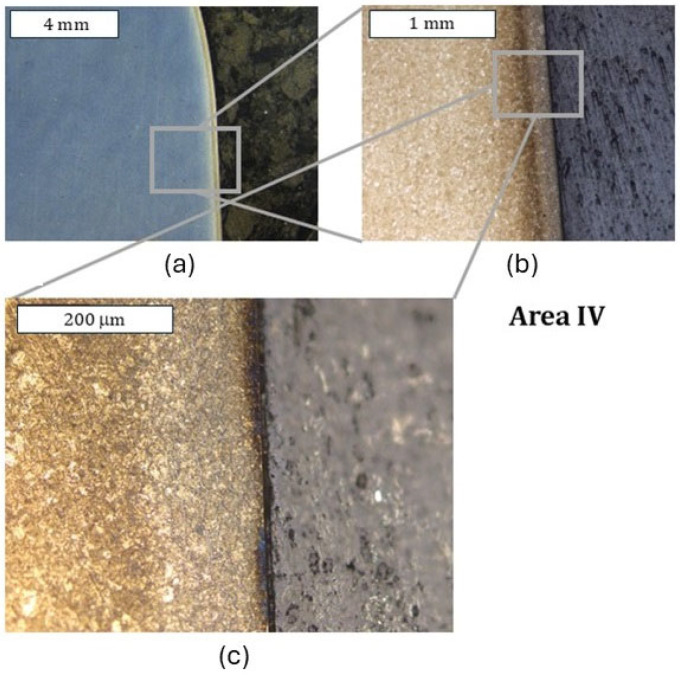
Macrostructure and microstructure of the die in zone IV: (**a**) stereoscopic microscopy; (**b**,**c**) light microscopy. Etched state.

During the microscopic observations, in the lower part of the die (zone IV), no clear degradation changes in the die were recorded. The observations conducted at higher magnifications demonstrated, however, that also in this area, surface oxidation took place; however, it was strongly limited (Figure 18). In this area, mechanical removal of the coating was also observed. It cannot be ruled out that the lack of the CrAlSiN coating or only its trace amounts are connected with the highest number of cycles worked over by the analyzed die. Smaller features of the substrate wear, and especially its oxidation, can be attributed to the lower thermal conductivity of CrAlSiN coatings, as indicated by the authors, caused by the presence of silicon in the chemical composition [36,37]. The authors assign this to better resistance to oxidation. This is also confirmed by Chang et al. [38].

The analyses carried out indicate that the decisive mechanism of destruction was abrasive wear. The authors in [39] indicate that the formation of a well-defined nanocomposite structure of CrAlSiN coatings is a factor that hinders typical mechanisms of deformation and mechanical damage. It is worth noting that during forging, the die surface temperature can exceed 500 °C. Polcar et al. [40] noted that the wear of CrAlSiN-type coatings was greater than at ambient temperature, and at 600 °C, severe degradation of the coating occurred. Similar observations were reported by Chan et al. [37]. However, the same studies showed that the wear rate decreases significantly at higher temperatures, i.e., 850 °C and 950 °C. The authors attribute this to the triboxidation phenomenon and the resulting formation of oxides that protect the coating against wear at these higher temperatures.

### 3.3. SEM Analysis

**Figure 19:**
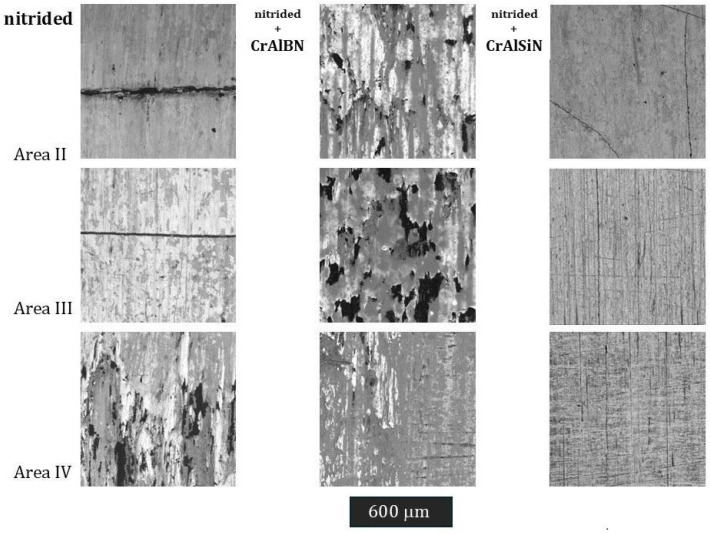
State of the surface of the examined tools in zones II to IV. SEM.

**Figure 20:**
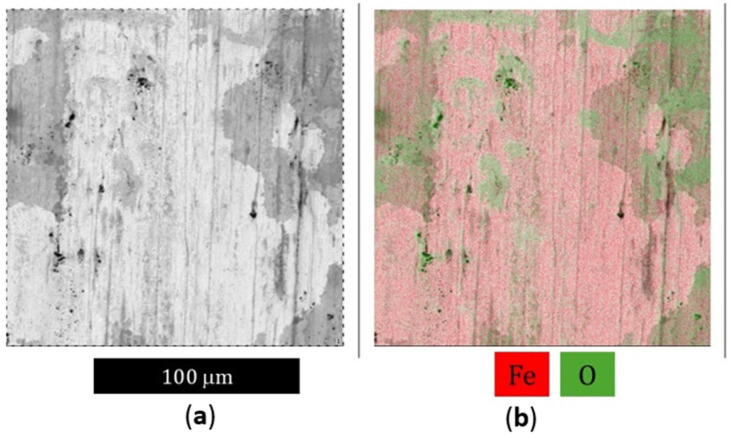
State of the surface of the nitrided tool (area III): (**a**) SEM image; (**b**) SEM image combined with the iron and oxygen distribution, SEM/EDX.

Also conducted were SEM microscopic observations of the tool surfaces in zones I to IV. The preliminary analysis showed no significant differences in zone I, and so the tests have been presented for zones II to IV (Figure 19). For these areas, the obtained results are similar to the microscopic observations on the cross-section. In the case of the nitrided layer in zone II, cracks transverse to the tool’s axis were observed. Also recorded were numerous areas where surface oxidation had occurred (Figure 20). It should be emphasized that the oxidation level was not high. As mentioned earlier, surface oxidation will be a factor contributing to the reduction in coating wear. In this case, the oxide layers that form will not protect the coating from wear. Due to the plastic deformation occurring, it was difficult to perform similar analyses in area III for the nitrided layer, as well as in the case of the die covered with the CrAlBN coating. There, the oxide layer mixed with the adhered material. In the case of the CrAlSiN coating, no oxygen-enriched areas were observed, which could indicate their at least partial oxidation. This is in line with the literature indications regarding the increased oxidation resistance of this coating.

In turn, for the die covered with the CrAlBN coating, in all the observed areas, numerous stick-ons were recorded, accompanied by plastic deformation. This proves a high tendency of the surface for the adhesion of the preform’s material to the surface of the die. In particular, when the adhesive bond formed is stronger than the grain boundary cohesion weakened by oxidation, oxidation is in turn promoted by the adhesion of the hot preform material to the die surface. A similar characteristic in terms of the changes was observed also in zone IV of the nitrided die. What is important is that such changes were not recorded in the case of the CrAlSiN coating. Therefore, a certain relationship can be observed concerning this area IV. In the case of the nitrided die, strong features of plastic deformation were noted (Figure 19), which resulted in severe wear (Figure 10d). In the case of the CrAlBN coating, plastic deformation was also observed, but to a lesser degree (Figure 14). In the case of the CrAlSiN coating, no features of plastic deformation were observed (Figure 19). Chang et al. [38] emphasize the role of plastic deformation in coating wear. High wear resistance is attributed to coatings with high resistance to plastic deformation. This can therefore explain the lowest wear of the CrAlSi coating in this area. In zone II, in the case of the CrAlBN-coated die, surface cracks were observed; however, they did not form a gap as in the case of the nitrided-only layer. They were also diversely directed.

The process of forging valves from nickel–chromium steel is characterized by extremely high stresses, leading to low tool durability. The conducted tests have shown that both applied surface engineering methods improved the life of the dies. However, it was also found that the best utility properties for the analyzed forming process are demonstrated by the die nitrided and simultaneously coated with CrAlSiN. This was attributed to more even wear, in which abrasive wear dominated, as well as lower susceptibility to plastic deformation and adhesion of the forged material.

This study presents the results of a performance analysis of three selected representative groups of forging tools (dies), for which surface engineering techniques were applied, aiming to increase their durability. The tools are used in a hot forging process of producing valve forgings from nickel–chromium steel (NCF3015), which results in the average durability of such tools being lower by one order of magnitude than that of the dies used to produce forgings from carbon steel. The dies in the analyzed forging process are made from hot work tool steel–chromium–molybdenum–vanadium steel W360, produced by Böhler. Normally, dies after thermal treatment are subjected to glow discharge nitriding. In order to increase their durability, beside the nitriding, for the two remaining groups, hybrid layers were also used. The hybrid treatment included a nitriding and a process of depositing PVD coatings. In this way, the second representative tool was obtained, a die with a nitrided layer and CrAlBN coating, whereas the third group was constituted by a tool with a nitrided layer and a CrAlSiN coating. All the examined tools introduced into the hot forging process characterized, as a result of their service life, in diversified wear. This unequivocally points to the fact that the selection of the surface engineering methods is of great importance to the durability of the tools participating in the analyzed process of forging elements from nickel–chromium steel.

## 4. Conclusions

Based on the realized detailed research study, mainly including a macroscopic analysis combined with 3D scanning, a microscopic analysis, and SEM tests, the following conclusions were drawn: It was established that the highest wear was observed in the case of the use of the nitrided-only tool (based on 3D scanning). The application of coatings made it possible to reduce the tool wear.

– In the case of the CrAlBN coating, no uniformity of wear was observed, which largely limits the possibility of predicting the durability of the tools participating in it. During the work of the tool, a local discontinuity of the coating took place, which favoured oxidation progressing along the grain boundaries of the tool material. During a longer service life, this will favour the spalling of the tool material, which can ultimately lead to the formation of losses with significant dimensions.
– The nitrided tool with a deposited CrAlSiN coating made it possible to produce the highest number of forgings, and the character of its wear was predictable, similarly to the nitrided tool. This coating also exhibited the lowest value of relative wear, understood as the loss of material referred to the number of forged elements. At the same time, the wear of the coating resulted in relatively uniform abrasion.
– In the case of the nitrided tool covered with the CrAlBN coating, similarly good results were obtained in the aspect of performance to those in the case of the CrAlSiN coating, as well as much better results compared to the standard tool, which had only been subjected to thermo-chemical treatment (nitriding). The tests showed no intensive wear in any of the zones, which is confirmed by the scanning results.
– The SEM analysis demonstrated that the CrAlBN coating favoured the adhesion of the preform’s material to the surface. This is probably the reason for the unpredictability of the die covered with this coating. The stick-ons of the preform material will favour the decohesion of the die material. This effect was not observed in the case of the CrAlSiN coating, which confirms the usefulness of the coating in the analyzed technological process.
– The tests demonstrated that the CrAlBN coating is less resistant to thermal effects (e.g., thermo-mechanical fatigue), which was partially confirmed by the slightly more intensively oxidating tool surface compared to the tool with the CrAlSiN coating.
– Future plans include further performance tests (aided by numerical modeling) for the proposed solutions, increasing the performance durability through the use of hybrid techniques, especially for longer production cycles and a larger number of tools, which will enable an even more thorough and complex analysis as well as provide the possibility for an economic analysis of the applied solutions, which will be optimal for the given tool used in the selected industrial process of hot die forging.

## Figures and Tables

**Table 1:** Chemical composition of the tested material serving as a base for the coating application.

Element	C	Si	Mn	P	S	Mo	Cr	Ni	V
Content	0.50	0.22	0.12	0.020	0.025	3.18	4.23	0.23	0.50

## Data Availability

The raw data supporting the conclusions of this article will be made available by the authors on request.
